# The role of parathyroid autotransplantation for hypoparathyroidism following total thyroidectomy with bilateral central neck dissection

**DOI:** 10.3389/fendo.2024.1402447

**Published:** 2024-07-03

**Authors:** Peisong Wang, Haowen Xue, Xuemei Zhu, Shuai Xue

**Affiliations:** General Surgery Center, Department of Thyroid Surgery, The 1st Hospital of Jilin University, Changchun, Jilin, China

**Keywords:** parathyroid autotransplantation, hypoparathyroidism, total thyroidectomy, central lymph node dissection, papillary thyroid carcinoma

## Abstract

**Background:**

Hypoparathyroidism is the most common complication for patients who undergo total thyroidectomy (TT) with bilateral central lymph node dissection (BCLND). The objective of this retrospective study was to investigate the relationship between parathyroid autotransplantation (PA) and postoperative hypoparathyroidism.

**Materials and Methods:**

Four hundred and sixty-five patients with papillary thyroid carcinoma (PTC) who underwent TT with BCLND (including prophylactic and therapeutic BCLND) by the same surgeon were enrolled in this retrospective study. They were divided into five groups based on the number of PAs. Group 0 was defined as no PA, while Group 1, 2, 3, and 4 were considered as 1, 2, 3, and 4 PAs during TT with BCLND, respectively.

**Results:**

Transient and permanent hypoparathyroidism occurred in 83 (17.8%) and 2 (0.4%) patients who underwent TT and BCLND, respectively. The incidence of transient hypoparathyroidism increased gradually with an increase in the number of PAs. Compared with the previous group, Groups 2 and 3 had significantly more cases of transient hypoparathyroidism (p=0.03 and p=0.04, respectively). All cases of permanent hypoparathyroidism occurred in the patients without PA. Compared with Group 0, there were more removed central lymph nodes (RCLNs) in patients with one PA. Furthermore, Group 2 had more metastatic central lymph nodes(MCLNs) and RCLNs than Group 1.The number of PAs was the only identified risk factor for transient hypoparathyroidism after the multivariate logistic regression analysis. The median parathyroid hormone level recovered to the normal range within 1 month after surgery.

**Conclusion:**

With an increasing number of PAs, the possibility of transient hypoparathyroidism also increases in patients with PTC who undergo TT and BCLND. Considering the rapid recovery of transient hypoparathyroidism in 1 month, two PAs during TT and BCLND could be a good choice, leading to an increase in the central lymph node yield and no permanent hypoparathyroidism. However, this conclusion should be validated in future multicenter prospective studies.

## Introduction

The number of thyroidectomies is significantly rising with the rapid increase in the global incidence of thyroid cancer (1, 2). Postoperative hypoparathyroidism, as one of the most common complications, remains a major concern for surgeons. Temporary and permanent hypoparathyroidism have been reported to occur approximately in 50% and 10% of patients who undergo total thyroidectomy (TT) with bilateral central lymph node dissection (BCLND) (3–5). Effectively identifying and protecting all parathyroid glands and their blood supply during bilateral thyroid surgery is particularly challenging due to anatomical variations in their positions and their blood vessels (6).

Consequently, an increasing number of experts have supported the autotransplantation of ischemic or unintentionally resected parathyroid glands into the muscle, which could significantly reduce the incidence of permanent hypoparathyroidism (7). Furthermore, numerous studies have indicated that the routine implantation of one or more parathyroid glands can effectively decrease the occurrence of permanent hypoparathyroidism following thyroidectomy (8, 9). However, the optimal number of parathyroid glands required for transplantation remains controversial. Some studies have suggested that the incidence of hypoparathyroidism is unrelated to the number of autotransplanted parathyroid glands, whereas other studies have reported conflicting results (10–12). This discrepancy is primarily due to variations in the extent of central lymph node dissection (CLND), which is an important confounding factor for hypoparathyroidism. Although all patients underwent TT, the extent of CLND had a significant impact on the blood supply to the parathyroid glands, particularly the inferior glands.

Therefore, the objective of this retrospective study, which enrolled patients who underwent TT with BCLND, was to investigate the relationship between parathyroid autotransplantation (PA) and postoperative hypoparathyroidism, and to provide clinicians with a theoretical basis for making decisions regarding PA.

## Material and methods

### Patients

All patients with papillary thyroid carcinoma (PTC) who underwent TT with BCLND (including prophylactic and therapeutic BCLND) by the same surgeon (PS Wang) in the Thyroid Surgery Department, General Surgery Center, First Hospital of Jilin University, from October 2020 to October 2022 were enrolled in this retrospective study. The inclusion criteria were as follows: (1) pathologically proven bilateral PTC, (2) TT with BCLND (with or without lateral neck dissection), (3) thyroid tumor or metastatic lymph nodes diagnosed preoperatively by fine needle aspiration (FNA), and (4) surgery completed by the same surgeon. The exclusion criteria were as follows: (1) reoperation and completion thyroidectomy, (2) age <18 years, (3) preoperative parathyroid gland dysfunction, and (4) follow-up duration <6 months. Finally, 465 patients were enrolled in this study and divided into five groups based on the number of PAs. Group 0 was defined as no PA, while Group 1, 2, 3, and 4 were considered as 1, 2, 3, and 4 PAs during TT with BCLND, respectively.

This study was approved by the Ethics Committee of the First Hospital of Jilin University (AF-IRB-032–06). Informed consent was obtained from all patients after they were informed that their clinical data would be used in this study.

### Surgical procedures

Suspicious thyroid and lymph node nodules were recommended to undergo FNA confirmation. Serum calcium and parathyroid hormone (PTH) were evaluated preoperatively. All the surgeries were performed by the same surgeon. Carbon nanoparticles (CN) are routinely used for parathyroid tissue identification. The CN injections and surgical procedures have been described in a previous study (13). During surgery, we meticulously dissected the thyroid capsule to identify and protect the parathyroid gland and its blood supply. In addition to the parathyroid color, the blood supply to the parathyroid glands was promptly assessed using a fine-needle pricking test (14). If blood oozed from the incision site, this was referred to as “good vascularity”. On the other hand, a pale parathyroid gland without blood oozing was immediately resected for PA. The excised specimens were carefully examined to identify parathyroid glands that may have been unintentionally removed. If an accidentally devascularized or resected parathyroid gland was found, PA to the contralateral sternocleidomastoid muscle pocket was performed immediately after intraoperative frozen section confirmation. PA was performed according to the guidelines (15).

### Postoperative treatment and follow-up

The recommendations for radioactive iodine ablation (RAI) and endocrine repressive therapy have been described in a previous study (16). Permanent hypoparathyroidism was defined as a decrease in serum PTH levels below the inferior normal limit (15 pg/ml) at 6 months postoperatively, regardless of hypocalcemic symptoms. Transient hypoparathyroidism was diagnosed if the decreased PTH level recovered within 6 months after surgery.

### Data collection

Data on the following clinicopathological characteristics were retrospectively collected: age, gender, body mass index (BMI), Hashimoto’s thyroiditis (HT), largest tumor diameter (LTD), tumor number, T stage of primary tumor, clinical metastatic lymph node (cN1), metastatic central lymph nodes (MCLNs), removed central lymph nodes (RCLNs), lateral lymph node metastasis (LLNM), identified parathyroid number, unintentional resected parathyroid number, transient and permanent hypoparathyroidism patients, and postoperative PTH level at the 1-day, 1-month and 6-month follow-ups.

### Statistical analysis

SPSS version 26 software (SPSS Inc., Chicago, IL, USA) was used for statistical analyses. Categorical data were expressed as absolute numbers and frequencies, and compared by the chi-square test or Fisher’s exact test. Continuous data with normal distribution was expressed as mean ± standard deviation (SD), while data with non-normal distributions was expressed as median with range. The t-test or Mann–Whitney U test was used for continuous data. The risk factors associated with transient hypoparathyroidism were ascertained by the multivariate logistic regression analysis, considering the variables that exhibited statistical significance in the univariate analysis. Statistical significance was defined as p<0.05.

## Results

### Clinicopathological characteristics of patients with TT and BCLND

**Figure 1:**
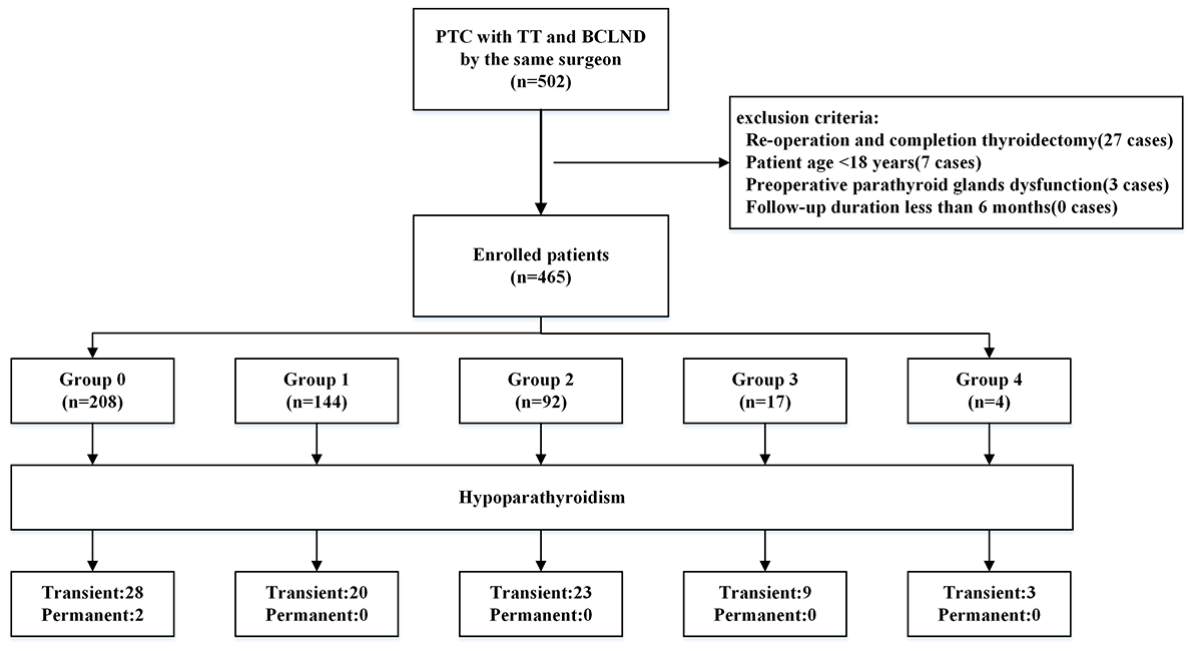
Flowchart of patient selection and grouping.

A total of 465 patients who underwent TT with BCLND were enrolled in our study (**Figure 1**). As shown in **Table 1**, average age was 47.3 ± 12.6 years and median BMI was 24.9 kg/m^2^, ranging from 15.7 to 37.9 kg/m^2^. Furthermore, 372 (80%) were female and 93 (20%) were male. Regarding the pathological characteristics, 309 (66.5%) patients had papillary thyroid microcarcinoma and the median LTD was 0.8 cm, ranging from 0.1 to 7.0 cm. In addition, 170 (36.6%) patients had HT and 77 (16.6%) were diagnosed with LLNM. With the help of CN, four parathyroid glands were identified in 389 (82.6%) patients, while three and two parathyroid glands were found in 80 (17.2%) and one (0.2%) patient, respectively. Moreover, unintended parathyroid resection was performed in 38 patients (8.2%).

**Table 1:** Clinicopathologic characteristics of all enrolled patients.

Variables	Patients (n=465)
Age, years (mean ± SD)	47.3±12.6
Gender Male (%) Female (%)	93 (20) 372 (80)
BMI (median [range])	24.9 [15.7-37.9]
HT Yes (%) No (%)	170 (36.6) 295 (63.4)
LTD,cm (median [range])	0.8 [0.1-7.0]
Tumor number (median [range])	3 [2-8]
T stage T1 (%) T2 (%) T3 (%) T4 (%)	394 (84.7) 24 (5.2) 28 (6.0) 19 (4.1)
cN1 Yes (%) No (%)	95 (20.4) 370 (79.6)
CLND MCLN (median [range]) RCLN (median [range])	0 [0-21] 9 [5-30]
LLNM Yes (%) No (%)	77 (16.6) 388 (83.4)
RAI Yes (%) No (%)	95 (20.4) 370 (79.6)
Parathyroid gland Identified number (median [range]) Resected number (median [range]) Implanted number (median [range])	4 [2-4] 0 [0-1] 1 [0-4]

SD, standard deviation; BMI, body mass index; HT, Hashimoto’s thyroiditis; LTD, largest tumor diameter; cN1, clinical metastatic lymph node; CLND, central lymph node dissection; MCLM, metastatic central lymph node; RCLN, removed central lymph node; LLNM, lateral lymph node metastasis; RAI, radioactive iodine ablation.

There were 208 (44.7%), 144 (31.0%), 92 (19.8%), 17 (3.7%), and 4 (0.8%) patients in Group 0, 1, 2, 3, and 4, respectively(**Table 2**). When comparing the clinicopathological characteristics, there were no significant differences in age, sex, BMI, HT, LTD, tumor number, T stage of the primary tumor, cN1, LLNM, RAI, identified parathyroid number, and unintentionally resected parathyroid number among the groups. Compared with Group 0, there were more RCLNs in patients with one PA (Group 1). Furthermore, Group 2 had more MCLNs and RCLNs than Group 1. Transient and permanent hypoparathyroidism occurred in 83 (17.8%) and 2 (0.4%) patients who underwent TT and BCLND, respectively. The incidence of transient hypoparathyroidism increased gradually with an increase in the number of PAs. Compared with the previous group, Groups 2 and 3 had significantly more cases of transient hypoparathyroidism (p=0.03 and p=0.04, respectively). All cases of permanent hypoparathyroidism occurred in the patients without PA.

**Table 2:** Clinicopathologic characteristics of patients according to the number of parathyroid autotransplantation.

Variables	Group 0 (n=208)	Group 1 (n=144)	Group 2 (n=92)	Group 3 (n=17)	Group 4 (n=4)	P^*^	P^#^	P^&^	P^^^
Age, years (mean ± SD)	46.7±11.4	47.9±11.3	46.8±13.1	43.8±9.4	44.0±13.0	0.31	0.42	0.21	0.92
Gender Male (%) Female (%)	41 (19.7) 167 (80.3)	26 (18.1) 118 (81.9)	20 (21.7) 72 (78.3)	5 (29.4) 12 (70.6)	1 (25.0) 3 (75.0)	0.70	0.49	0.70	1.00
BMI (median [range])	24.8 [19.5-37.3]	25.2 [17.1-37.0]	24.4 [15.7-37.9]	25.1 [21.7-32.4]	24.3 [23.0-26.3]	0.72	0.53	0.37	0.36
HT Yes (%) No (%)	74 (35.6) 134 (64.4)	57 (39.6) 87 (60.4)	31 (33.7) 61 (66.3)	6 (35.3) 11 (64.7)	2 (50.0) 2 (50.0)	0.45	0.36	0.90	0.62
LTD,cm (median [range])	0.7 [0.1-5.0]	0.8 [0.2-7.0]	0.9 [0.2-5.5]	0.8 [0.4-4.8]	0.9 [0.6-1.2]	0.15	0.34	0.42	0.64
Tumor number (median [range])	3 [2-8]	3 [2-8]	3 [2-7]	3 [2-8]	2 [2-5]	0.33	0.79	0.62	0.52
T stage T1 (%) T2 (%) T3 (%) T4 (%)	186 (89.4) 10 (4.8) 6 (2.9) 6 (2.9)	118 (81.9) 9 (6.3) 10 (6.9) 7 (4.9)	77 (83.8) 4 (4.3) 7 (7.6) 4 (4.3)	10 (58.8) 1 (5.9) 5 (29.4) 1 (5.9)	3 (75.0) 0 (0) 0 (0) 1 (25.0)	0.18	0.93	0.11	0.30
cN1 Yes (%) No (%)	33 (15.9) 175 (84.1)	27 (18.8) 117 (81.2)	25 (27.2) 67 (72.8)	8 (47.1) 9 (52.9)	2 (50%) 2 (50%)	0.48	0.13	0.10	1.00
CLND MCLN (median [range]) RCLN (median [range])	0 [0-21] 8 [5-29]	0 [0-21] 9 [5-30]	1 [0-18] 11 [5-25]	2 [0-13] 13 [5-26]	2 [0-15] 11 [6-29]	0.55 0.04	0.01 0.04	0.61 0.21	0.83 1.00
LLNM Yes (%) No (%)	23 (11.1) 185 (88.9)	23 (16.0) 121 (84.0)	22 (23.9) 70 (76.1)	8 (47.1) 9 (52.9)	1 (25.0) 3 (75.0)	0.18	0.13	0.07	0.60
RAI Yes (%) No (%)	40 (19.2) 168 (80.8)	29 (20.1) 115 (79.9)	19 (20.7) 73 (79.3)	6 (35.3) 11 (64.7)	1 (25.0) 3 (75.0)	0.83	0.92	0.21	1.00
Parathyroid gland Identified number (median [range]) Resected number (median [range])	4 [2-4] 0 [0-1]	4 [3-4] 0 [0-1]	4 [3-4] 0 [0-1]	4 [4-4] 0 [0-1]	4 [4-4] 0 [0-0]	0.17 0.27	0.24 0.28	0.44 0.32	1.00 0.90
Hypoparathyroidism Transient (%) Permanent (%)	28 (13.5) 2 (1.0)	20 (13.9) 0 (0)	23 (25) 0 (0)	9 (52.9) 0 (0)	3 (75) 0 (0)	0.91 NA	0.03 NA	0.04 NA	0.60 NA
Postoperative PTH (pg/ml) 1 day (median [range]) 1 month (median [range]) 6 months (median [range])	48.1 [0-136.1] 45.4 [0-98.7] 46.7 [0-88]	47.2 [0-155.4] 52.6 [10.4-124] 66.1 [29.4-95.4]	34.7 [0-91.9] 45 [19.1-149.6] 49.4 [19.5-85.9]	12.7 [0-91.4] 44.1 [26-86] 56.6 [14.8-87]	0 [0-16] 33 [16.9-45.8] 35 [22.8-48]	0.87 0.77 0.18	0.02 0.61 0.21	0.00 0.99 0.31	0.05 0.09 0.07

SD, standard deviation; BMI, body mass index; HT, Hashimoto’s thyroiditis; LTD, largest tumor diameter; cN1, clinical metastatic lymph node; CLND, central lymph node dissection; MCLM, metastatic central lymph node; RCLN, removed central lymph node; LLNM, lateral lymph node metastasis; RAI, radioactive iodine ablation.

*:Group 0 vs Group 1.

#:Group 1 vs Group 2.

&:Group2 vs Group 3.

^:Group 3 vs Group 4.

### Risk factors and recovery for transient hypoparathyroidism

**Figure 2:**
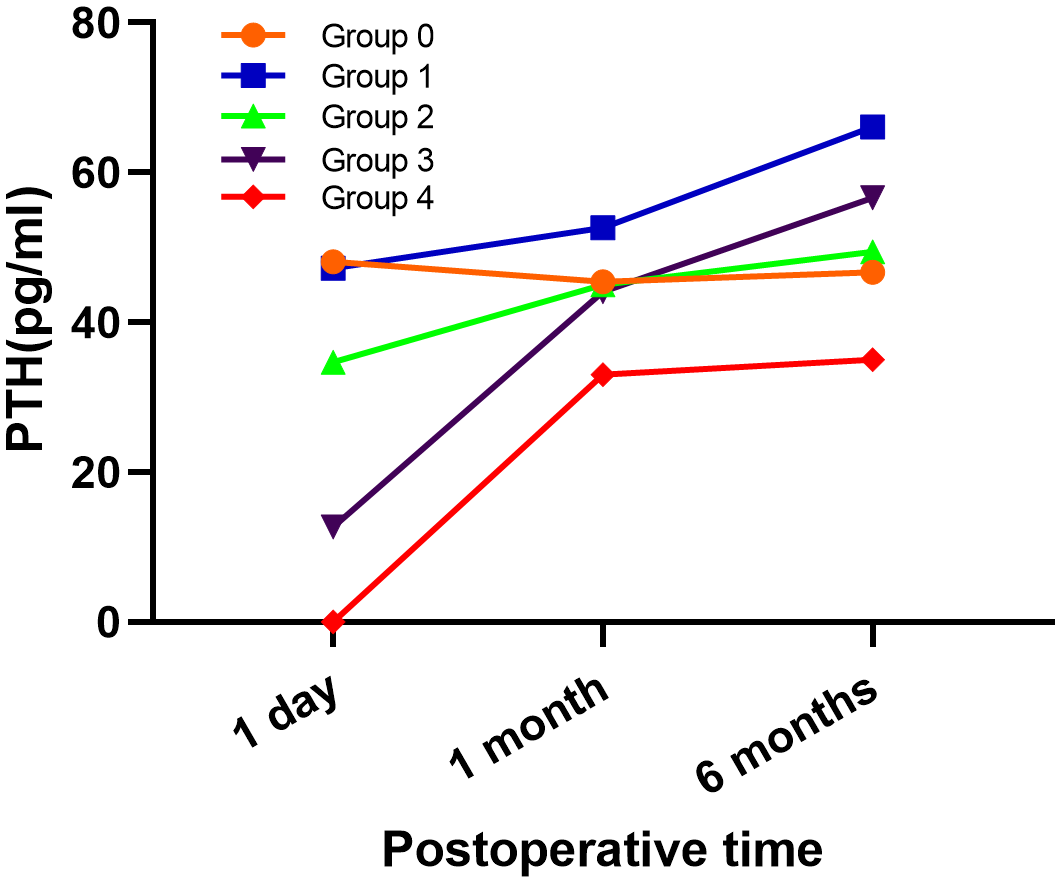
Pattern of PTH recovery for different group.

As shown in **Table 3**, the univariate analysis of the clinicopathological characteristics revealed that younger age, LLNM and higher PA were associated with transient hypoparathyroidism. However, the number of PAs was the only identified risk factor for transient hypoparathyroidism after the multivariate logistic regression analysis (**Table 4**). After a 6-month follow-up, the percentage of serum PTH levels that had recovered to the normal range was 99.0%, 100%, 100%, 100%, and 100% in the five groups, respectively. As shown in **Figure 2**, the recovery patterns of the different groups were almost the same, and the median PTH level recovered to the normal range 1 month after surgery. Notably, the PTH concentrations of four patients in Group 4 with four PAs also recovered to normal within 1 month.

**Table 3:** Univariate analysis of clinicopathologic characteristics affecting to transient hypoparathyroidism.

Variables	Normal (n=382)	Transient (n=83)	P
Age, years (mean ± SD)	48.1±14.5	43.7±11.1	0.01
Gender Male (%) Female (%)	73 (19.1) 309 (80.9)	20 (24.1) 63 (75.9)	0.30
BMI (median [range])	24.1 [15.7-37.9]	25.4 [15.8-35.7]	0.13
HT Yes (%) No (%)	137 (35.9) 245 (64.1)	33 (39.8) 50 (60.2)	0.50
LTD,cm (median [range])	0.8 [0.1-5.5]	0.8 [0.2-7]	0.25
Tumor number (median [range])	3 [2-8]	3 [2-6]	0.23
T stage T1 (%) T2 (%) T3 (%) T4 (%)	329 (86.1) 17 (4.5) 22 (5.8) 14 (3.6)	65 (78.3) 7 (8.4) 6 (7.2) 5 (6.1)	0.33
cN1 Yes (%) No (%)	74 (19.4) 308 (80.6)	21 (25.3) 62 (74.7)	0.23
CLND MCLN (median [range]) RCLN (median [range])	0 [0-21] 9 [5-30]	0 [1-21] 10 [5-30]	0.07 0.06
LLNM Yes (%) No (%)	56 (14.7) 326 (85.3)	21 (25.3) 62 (74.7)	0.02
RAI Yes (%) No (%)	79 (20.7) 303 (79.3)	16 (19.0) 67 (81.0)	0.77
Parathyroid gland Identified number (median [range]) Resected number (median [range]) Implanted number (median [range])	4 [2-4] 0 [0-1] 1 [0-4]	4 [3-4] 0 [0-1] 1 [0-4]	0.10 0.19 0.00

SD, standard deviation; BMI, body mass index; HT, Hashimoto’s thyroiditis; LTD, largest tumor diameter; cN1, clinical metastatic lymph node; CLND, central lymph node dissection; MCLM, metastatic central lymph node; RCLN, removed central lymph node; LLNM, lateral lymph node metastasis; RAI, radioactive iodine ablation.

**Table 4:** Multivariate analysis of clinicopathologic characteristics affecting to transient hypoparathyroidism.

Variables	P value	Odds ratio (CI)
Age, years (mean ± SD)	0.09	0.970 (0.912-1.142)
Gender	0.75	0.924 (0.757-1.568)
BMI (median [range])	0.85	1.245 (0.822-1.312)
HT	0.94	0.775 (0.593-1.764)
LTD,cm (median [range])	0.17	1.245 (0.829-1.449)
Tumor number (median [range])	0.56	1.185 (0.756-1.342)
T stage T1 (%) T2 (%) T3 (%) T4 (%)	0.59 0.74 0.42 0.44	1.454 (0.245-5.752) 3.254 (0.389-14.223) 0.754 (0.335-2.389)
cN1	0.79	1.019 (0.921-1.754)
CLND MCLN (median [range]) RCLN (median [range])	0.55 0.68	0.994 (0.877-1.268) 1.241 (0.785-1.599)
LLNM	0.11	0.985 (0.654-2.386)
RAI	0.91	1.324 (0.987-2.685)
Parathyroid gland Identified number (median [range]) Resected number (median [range]) Implanted number (median [range])	0.16 0.77 0.00	1.985 (0.854-3.574) 1.856 (0.549-2.996) 2.171 (1.863-3.964)

CI, confidence interval; SD, standard deviation; BMI, body mass index; HT, Hashimoto’s thyroiditis; LTD, largest tumor diameter; cN1, clinical metastatic lymph node; CLND, central lymph node dissection; MCLM, metastatic central lymph node; RCLN, removed central lymph node; LLNM, lateral lymph node metastasis; RAI, radioactive iodine ablation.

## Discussion

Hypoparathyroidism, especially permanent hypoparathyroidism, is the most common complication of thyroidectomy and has a negative impact on the quality of life and mortality of patients (17, 18). PA has proven to be an effective method for preventing permanent hypoparathyroidism (19); however, it remains controversial whether routine PA should be performed. In addition, the recommended number of PAs is not well understood. These conflicting data were mainly due to confounders, such as the extent of thyroidectomy and CLND, surgeon experience, and follow-up time. Subsequently, patients who underwent TT and BCLND performed by the same surgeon with a 6-month follow period were enrolled in this study. We found that the number of PAs was the only risk factor for transient hypoparathyroidism, which resolved within 1 month. Moreover, we shared information on four patients with four PAs. The PTH levels of all patients recovered to within the normal range and permanent hypoparathyroidism occurred only in patients without PA.

CLND has been verified to be associated with hypoparathyroidism. Some studies have demonstrated that prophylactic CLND does not provide benefits for recurrence and mortality, which causes hypoparathyroidism (20). In recent years, mounting evidence has revealed that RCLNs are associated with PTC recurrence. A retrospective study from China found that a central lymph node yield (CLNY) <11 was an independent risk factor for recurrence and persistence in patients with PTC (21). Another study from the United States also demonstrated that patients with PTC and a lower CLNY (average, 2.5) experienced a higher rate of recurrence than those with a higher CLNY (average, 10.3) (22). CLND makes parathyroid protection even more challenging. Distinguishing the parathyroid gland from the lymph node, fatty tissue, or thymus, in addition to protecting the tiny blood vessels that supply the parathyroid gland, make preservation of the parathyroid *in situ* much more difficult. PA is usually inevitable following BCLND. According to our results, patients with two PAs presented with more RCLNs (median, 11) and MCLNs (median, 1), which was associated with reduced recurrence according to the publications mentioned above; However, this finding should be verified with a longer follow-up in our study. Moreover, cases with two PAs had a higher incidence of transient hypoparathyroidism (25%), which is consistent with the previous literature (19). Appropriately managed transient hypoparathyroidism does not have a substantial negative impact on the patients’ quality of life (23). The patients recovered within 1 month, and no permanent hypoparathyroidism occurred in our study. According to this evidence, two PAs for patients with TT and BCLND could be a good choice, resulting in a higher CLNY and relatively less hypoparathyroidism.

Numerous novel methods exist for parathyroid identification, such as near-infrared autofluorescence and CN (13, 24). The most difficult aspect of parathyroid protection is the preservation of the tiny blood vessels. Indocyanine green fluorescence can only be used to predict parathyroid blood supply postoperatively (25). In our study, the rate of permanent hypoparathyroidism (2/465, 0.4%) was lower than that reported in previous studies (19). We summarize the possible reasons as follows: (1) CN helped us identify the parathyroid gland. As previously reported, CN can increase the CNLY and decrease unintended parathyroid resection (13). (2) We carefully checked the specimens. After the thyroid was removed, the specimen was cut into pieces to identify the intrathyroidal parathyroid glands. Thus, we decreased unintended parathyroid resection to the greatest extent. (3) We promptly evaluated the blood supply of the parathyroid gland with the help of parathyroid color and a fine-needle pricking test. If the parathyroid color is normal or dark, and blood was oozing out from the incision, good vascularity was indicated. Otherwise, if the parathyroid was pale, and blood did not ooze out after fine-needle pricking, we immediately resected it for PA. (4) We implanted in a dispersed manner. Each parathyroid gland was implanted in at least two pockets of the sternocleidomastoid muscle for complete contact.

In our cohort, prophylactic CLND was performed for 370 patients while therapeutic CLND for 95 cases. Among 38 patients with unintended parathyroid resection, 29 cases (7.8%, 29/370) were found among prophylactic CLND group while 9 cases (9.5%, 9/95) for therapeutic CLND group. Moreover, one case with permanent hypoparathyroidism was identified in prophylactic CLND group while another permanent hypoparathyroidism in therapeutic CLND group. For transient hypoparathyroidism, there were 66 cases (17.8%,66/370) in patients with prophylactic CLND and 17 cases (17.9%,17/95) in therapeutic CLND group. As previously reported, the devascularization of the parathyroid glands during thyroidectomy is related to the extent of thyroid surgery (3–5). During CLND, surgeons will be more likely to dissect central lymph nodes aggressively during therapeutic CLND. Therefore, parathyroid preservation will be more difficult. It is expected higher devascularization of the parathyroid glands or more unintended parathyroid resection in therapeutic group compared to prophylactic CLND. However, the incidence of unintended parathyroid resection, transient and permanent hypoparathyroidism do not seem to be different significantly between two CLND group as expected. In our cohort, CLND regardless of prophylactic or therapeutic,was all performed to remove of all nodes and fibro-fatty tissue extending laterally from the medial border of the common carotid artery to the midline of the trache and vertically from the hyoid bone to the thoracic inlet. We will not compromise the extent of CLND even when it’s prophylactic. Moreover, we have realized therapeutic CLND and LLNM might be important factors for parathyroid protection. Because of that, factors like cN1 and LLNM were included and performed statistical analysis. These factors in our cohort were not identified to be related with hypoparathyroidism. This results in our study should be validated in future multicenter prospective studies.

Firstly, this was a retrospective study based on the experience of a tertiary medical center, which may have led to a selection bias. Secondly, several potential risk factors, such as tumor location and specific PA conditions (superior or inferior parathyroid gland), were not investigated. In addition, the recurrence rates in each group were not compared because the follow-up period was relatively short. Finally, PA for patients with TT and BCLND may be a good choice, resulting in an increased CLNY and relatively less hypoparathyroidism according to our study results. Whether the two routine PAs are recommended remains controversial and needs to be validated through multicenter prospective studies.

## Conclusion

With an increasing number of PAs, the possibility of transient hypoparathyroidism also increases in patients with PTC who undergo TT and BCLND. Considering the rapid recovery of transient hypoparathyroidism in 1 month, two PAs during TT and BCLND could be a good choice, leading to an increase in the CLNY and no permanent hypoparathyroidism. However, this conclusion should be validated in future multicenter prospective studies.

## Data availability statement

The raw data supporting the conclusions of this article will be made available by the authors, without undue reservation.

## Ethics statement

The studies involving humans were approved by the ethics committee of the First Hospital of Jilin University. The studies were conducted in accordance with the local legislation and institutional requirements. The participants provided their written informed consent to participate in this study.

## Author contributions

PW: Conceptualization, Investigation, Methodology, Resources, Visualization, Writing – original draft. HX: Methodology, Software, Validation, Writing – original draft. XZ: Investigation, Methodology, Software, Validation, Visualization, Writing – original draft. SX: Conceptualization, Methodology, Supervision, Visualization, Writing – review & editing.
